# The Sunlight Cure

**Published:** 1892-04-30

**Authors:** 


### The Hospital

An Institutional Journal of

**Science, Medicine, Nursing, and Philanthropy.**

*For Hospitals, Asylums, Infirmaries, Dispensaries, Medical Practitioners, Students, Nurses, and the Charitable Public.*

Vol. XII.—No. 292.] [April 30, 1892.

### The Sunlight Cure.

Medical men have long been credited with being the most prejudiced of all civilised mankind. There may have been some truth in that view of the case in past times. But however it was in the days of our professional predecessors it is certain that no class of men of our own time are more accessible to new ideas than doctors, and there are perhaps none who take such an infinity of strenuous pains in the discovery of new truth which may be available for practical purposes. *The Practitioner* is one of the soberest of professional journals, and its three distinguished editors are not only like Cæsar's wife, above the possibility of suspicion, but they would all be instantaneously and unanimously trusted by every educated member of the medical profession. And yet it is *The Practitioner* which is the first to bring "The Sunlight Cure" before the notice of the medical profession, even though that "cure" has been developed and elaborated by a man who is not a member of the medical profession.

What is "The Sunlight Cure"? In the first place, it seems to be a cure of a real and actual character. In the second place it is a cure which has no mystery about it. We are not asked to accept a fluid or a solid of an unknown nature on the authority of a man of uncertain character. The whole system is plain and above board. The cure is carried out at the village of Veldes, which is situated in one of the Austrian provinces lying between the Julian Alps and the Karawanks. It nestles on the shores of a beautiful lake in the hills, 1,600 feet above the level of the sea. The village is protected from the north and east winds; the climate is mild and dry; there are abundant facilities in the surrounding district for walking, driving, boating, fishing and sport.

With such an environment "cures" of almost any kind would have an excellent chance of success. It speaks volumes for Herr Rikli's good sense that he has selected so admirable a situation for his clinical experiments. An account of the proceedings of a single day will put readers in possession of the cure. To begin with; the patient rises with the sun, which is often as early as four o'clock in the morning. The earliest business of the day is to take his first "air bath." For this purpose he is provided with a quart of fresh milk in a flask, six ounces of whole-meal bread, and a jar of honey. Lightly clothed, he sets out for the foot of a neighbouring hill, on the summit of which his air bath is to be taken. Before ascending the hill he removes his shoes and stockings, and commences the ascent with bare feet. At that early hour the grass is usually wet with dew, and the pine woods through which he passes are not yet penetrated by the morning sun. Notwithstanding this, as he ascends the hill he must remove his other articles of clothing, one by one, until he appears at the top of the ascent as Adam appeared in the garden of Eden—in a state of absolute nudity. In the case of a very squeamish person a handkerchief suspended from a belt or a pair of bathing drawers is allowed. But most of the patients find it convenient to dispense with covering altogether.

In this natural costume, breakfast, which consists of the quart of fresh milk and the six ounces of whole-meal bread with honey already mentioned, is partaken of; and then the air bath properly begins. It consists of nothing more than walking, running, playing at various games, or reading for two or three hours under the morning sun in a state of absolute nudity. The picture is primitive to the very last degree. We are carried back in imagination from the stifling laboratory of the physiological experimenter in stifling London to a period and to conditions and methods anterior to all civilisation. Who shall say after this that the cultured doctor of the nineteenth century is destitute of a philosophic mind? Even Mathew Arnold would have exempted British medical men from his universal accusation of "inaccessibility to new ideas."

But to return to our sunlight patients, whom we left dancing and tearing about in a condition of unprotected nature. After two or three hours of various exercise the summit of the hill is left in the distance, and the patient makes his way to a "hut" on the shores of the lake we have mentioned. Here he takes his first "sun bath." This is really a Turkish bath in the open air, in which the blazing sun is the only heating apparatus. The whole of the surface of the body is exposed to the heat of the midday sun. The patients lie in a row in the open air, protected from public view by a screening fence. The head alone is defended against the full glare of the sun. From twenty to sixty minutes is occupied in this "roasting" process, and the patient is turned, or rather turns himself, in all directions so as to completely expose every part of the surface of the body to the action of the light. The roasting over, he is wrapped in thick blankets and left in the sun for a further period of ten minutes, with the object of bringing on a free perspiration. This done, he is carried to a steam or water bath, and there vigorously manipulated by an attendant after the Turkish bath fashion.

This ends what may be considered the proceedings of the morning. The next thing to be done is to prepare for the midday meal, and this preparation may consist of a short walk or a rest as the patient feels disposed. The lunch is of the same frugal character as the breakfast; it includes whole meal or rye bread, stewed fruit, honey, milk sour or fresh, and in the case of the hungry Englishman, a lightly boiled fresh egg by way of special luxury. In the afternoon a second sun bath is taken; and this is in all respects similar to the first, except that it occupies about half the time. After the bath the patient goes for a walk "with bare feet," a proceeding which is enjoyed by a good many; and then follows dinner, the principle meal of the day. This consists of bread, vegetables, and fruit; but meat, alcohol, and butter are prohibited except in special cases. After dinner the patients are free to amuse themselves in their own way; but at sunset, everybody must go to bed. It is easy to believe that a long day spent in the open air will dispose most of the visitors to go to bed even before "the curfew tolls the knell of parting day."

That, without some modification, is the process: what are the results? The writers in the article of *The Practitioner*, for there are two of them, are both medical men, and it is clear, therefore, that in stating their conclusions they are free from the suspicion of having any "axe to grind." On the contrary, their not unnatural prejudices would dispose them to an attitude of sceptical criticism. But, with praiseworthy candour, they rise superior to every temptation to be unfair. In their own words, "After allowing a liberal discount for sources of error, it is not possible for an impartial observer on the spot to doubt that many invalids who visit Veldes undergo a marked change for the better. This fact is attested not only by their own assertions or by the opinions of their attendants, or even by the relief of evident symptoms, but also by a rapid improvement in their appearance, an increase of appetite and vigour, and the unmistakeable look of health which they present. It is asserted that only three deaths have occurred during the last thirty years." The writers point out that Veldes enjoys a beautiful climate, remote from the worries of civilisation, and that it has every natural advantage which can be well imagined. But they decidedly incline to the opinion that the sun baths have a powerful curative effect upon those who are exposed to them. There is a qualified medical man working in conjunction with Herr Rikli, and he, as well as the attendants and most of the patients, are firmly convinced of the decided efficacy of sunlight in the production of curative results. The writers remind us of the fact that the physiological effects of sunlight on the growth of plants and animals are familiar to everybody. "Rapid growth, a blanched appearance, due to a deficiency of chlorophyll, together with imperfect development, are the well-known effects of depriving plants of light. Conditions, which would seem to be in some respects analogous, are observed among the pallid and sickly inhabitants dwelling in the narrow courts of cities and in dark mountain valleys. The striking change produced in pale, town-bred children after a few days exposure to the sun in the fields or by the sea is accepted as a sort of measure of the benefit received from the change; and these changes must depend on light, as they are not produced by any other factors, and it is a common experience that the effect is greater in bright than in dull weather." This reasoning is so obviously just that the most unscientific cannot fail to be impressed by it. The principal conclusion to be drawn therefrom is that everybody should make the fullest possible use of our somewhat limited supply of sunshine, both by living in the open air, and by exposing every room in his house to the direct rays of the sun as much as possible.

